# Effect of *Helicobacter pylori* eradication on gastric cancer risk in patients with intestinal metaplasia or dysplasia: a meta-analysis of randomized controlled trials

**DOI:** 10.3389/fmicb.2025.1530549

**Published:** 2025-03-12

**Authors:** Qiang Fu, Huidong Yu, Ming Liu, Liang Chen, Weiyang Chen, Ziyi Wang, Wenya Li

**Affiliations:** ^1^Department of Thoracic Surgery, The First Hospital of China Medical University, Shenyang, China; ^2^State Key Laboratory of Molecular Oncology, National Cancer Center, National Clinical Research Center for Cancer, Cancer Hospital, Chinese Academy of Medical Sciences and Peking Union Medical College, Beijing, China; ^3^Section of Esophageal and Mediastinal Oncology, Department of Thoracic Surgery, National Cancer Center, National Clinical Research Center for Cancer, Cancer Hospital, Chinese Academy of Medical Sciences and Peking Union Medical College, Beijing, China

**Keywords:** *Helicobacter pylori*, gastric cancer, intestinal metaplasia, dysplasia, precursor, cancer risk, meta-analysis

## Abstract

**Background:**

Observational studies suggest that *Helicobacter pylori* (*H. pylori*) is associated with an increased risk of gastric cancer, yet the effect of *H. pylori* eradication on gastric cancer risk in patients with intestinal metaplasia (IM) or dysplasia remains controversial. The purpose of this study was to summarize the evidence from randomized controlled trials (RCTs) investigating *H. pylori* eradication on gastric cancer risk in patients with IM or dysplasia to determine the evidence base.

**Methods:**

PubMed, Embase, Cochrane Library, Web of science and China National Knowledge Internet database were searched for RCTs published through May 2024 in adults with IM or dysplasia comparing the risk of gastric cancer following *H. pylori* eradication versus no eradication therapy. Relative risk (RR) with its 95% confidence interval (CI) using random-effects model were employed for the effect estimate. Sensitivity, meta-regression, and subgroup analyses were also calculated.

**Results:**

Sixteen RCTs involving 15,027 patients with IM or dysplasia met the inclusion criteria. In a pooled analysis, *H. pylori* eradication resulted in a 45% reduction in RR for gastric cancer risk relative to no eradication (RR: 0.55; 95% CI: 0.46–0.67; *p* < 0.001). *H. pylori* eradication significantly reduced the risk of gastric cancer in patients with dysplasia (RR: 0.51; 95% CI: 0.32–0.82; *p* = 0.005), and IM (RR: 0.61; 95% CI: 0.40–0.93; *p* = 0.022). Further, if the study conducted in countries other than those in Asia, sample size <500, percentage of male <50.0%, follow-up duration <5.0 years, and low study quality, then there was no significant association between *H. pylori* eradication and a decreased risk of gastric cancer.

**Conclusion:**

*H. pylori* eradication is protective against gastric cancer in patients with IM or dysplasia.

**Systematic review registration:**

INPLASY202530010, https://inplasy.com/.

## Introduction

Gastric cancer remains to rank fifth for incidence and fourth for mortality globally (Torre et al., 2015; Ferlay et al., 2010; Ferlay et al., 2013). Asian countries have a higher incidence of gastric cancer compared with Western countries (Torre et al., 2015). *Helicobacter pylori* (*H. pylori*) infection is known to play an important role in the progression of gastric cancer, especially for intestinal type gastric cancer (Parsonnet et al., 1991; Huang et al., 2003; Maeda et al., 2000; Correa et al., 1990; International Agency for Research on Cancer, 1994; Uemura et al., 2001). Patients with superficial gastritis are often seen to progress from atrophy to dysplasia to intestinal-type gastric cancers, and it has been demonstrated that *H. pylori* infection can cause chronic gastritis, gastric atrophy, dysplasia, and gastric cancer (Correa, 1984; Correa, 1988). However, the use of *H. pylori* eradication for preventing gastric cancer in patients with intestinal metaplasia (IM) or dysplasia has not consistently been shown to be beneficial, Some studies contend that when the lesion has advanced to the stage of IM or dysplasia, even if *H. pylori* is eliminated, the cellular and tissue alterations previously triggered by it might be irreversible. Recently, *H. pylori* eradication was recommended following endoscopic resection for patients with early gastric cancer to prevent the progression to metachronous gastric cancer, but its effect on the residual risk of gastric cancer in patients with IM or dysplasia has not been explored (Asaka et al., 2010; Malfertheiner et al., 2012; Choi et al., 2018). A previous meta-analysis indicated that *H. pylori* eradication is associated with lower risk of gastric cancer (Fuccio et al., 2009). Another recent meta-analysis has suggested that *H. pylori* eradication may be an effective means for primary and secondary prevention of gastric cancer (Lee et al., 2016), while a different important meta-analysis based on randomized controlled trials (RCTs) found that patients with IM or dysplasia did not benefit from *H. pylori* eradication (Chen et al., 2016). The latest meta-analysis indicates that *H. pylori* can significantly prevent the progression of intestinal metaplasia and improve chronic atrophic gastritis and intestinal metaplasia (Zhu et al., 2023). The contradictory findings from these studies have opened arguments on whether *H. pylori* eradication could reduce the risk of gastric cancer in patients with a precancerous condition. Additionally, several RCTs were not completed searched and these studies have been excluded. This meta-analysis updates these previous publications and aims to provide more comprehensive results for the effect of treating *H. pylori* infection for preventing gastric cancer. Further, whether this relationship is differing according study or patients’ characteristics are also evaluated.

## Methods

### Search strategy and selection criteria

This review was conducted and reported according to the Preferred Reporting Items for Systematic Reviews and Meta-Analysis Statement issued in 2009 (Checklist S1) (Moher et al., 2010). The electronic databases of PubMed, Embase, Cochrane Library, Web of science and China National Knowledge Internet database were systematically searched for articles published through May 2024 using [“*Helicobacter pylori*” OR (“helicobacter” AND “pylori”) OR “helicobacterpylori”) AND (“stomach neoplasms” OR (“stomach” AND “neoplasms”) OR “stomach neoplasms” OR (“gastric” AND “cancer”) OR “gastric cancer”] as core search terms. Additional potential included trials were searched for using the aforementioned terms on: http://www.clinicaltrials.gov, which registers trials that are completed but not yet published. Finally, manual searches of the reference lists of all relevant original and review articles were conducted to identify additional eligible studies.

The literature search was undertaken by 2 authors independently and any inconsistencies were settled by group discussion until a consensus was reached. A study was eligible for inclusion if the following criteria were met: (1) Design: the study should design as RCT; (2) Precursor status: all of the included patients had IM or dysplasia; (3) Intervention: patients either did or did not receive *H. pylori* eradication; and (4) Outcomes: the incidence of gastric cancer was reported. The exclusion criteria included: (1) patients with other Precursor status; (2) the study with observational design; (3) both intervention and control group received *H. pylori* eradication; and (4) the incidence of gastric cancer was not available or calculated.

### Data collection and quality assessment

Two reviewers independently extracted all data, with disagreements resolved in consultation with a third investigator. The following items were extracted from the included articles: first author, country, sample size, mean age, Precursor status, percentage male, *H. pylori* diagnosis, number of gastric cancer cases, intervention, follow-up duration, and study design subscales. The study quality was assessed using the Jadad scale (1), which is based on randomization (1 or 0), concealment of the treatment allocation (1 or 0), blinding (1 or 0), completeness of follow-up (1 or 0), and the use of intention-to-treat analysis (1 or 0). In this meta-analysis, a study with a score of 4 or greater was regarded as high quality (Jadad et al., 1996).

### Statistical analysis

The incidence of gastric cancer was presented as frequencies and percentages. The pooled relative risk (RR) and 95% confidence interval (CI), as well as the heterogeneity of the included studies, were computed using random-effect (DerSimonian and Laird) models (DerSimonian and Laird, 1986; Ades et al., 2005). Heterogeneity between studies was investigated using the Q statistic, and *p* < 0.10 were indicative of significant heterogeneity (Deeks et al., 2008; Higgins et al., 2003). Each trial was sequentially excluded to carry out a sensitivity analysis to assess the influence of each single study on the meta-analysis (Tobias, 1999). In addition, to investigate the potential heterogeneity between RCTs, a meta-regression was performed based on publication year, sample size, mean age, percentage male, and follow-up duration (Thompson and Higgins, 2002). Subsequently, subgroup analyses were conducted for gastric cancer studies according to country, publication year, sample size, mean age, percentage male, Precursor status, *H. pylori* diagnosis, follow-up duration, and study quality. Ratios and *p*-values between subgroups were calculated using the Chi-square test and meta-regression (Deeks et al., 2001). Publication bias was evaluated using funnel plots and Egger’s (Egger et al., 1997) and Begg’s tests (Begg and Mazumdar, 1994), with *p* < 0.05 considered to indicate significant publication bias. Two-tailed *p* < 0.05 were considered statistically significant. All statistical analyses were performed with STATA 10.0 software (Stata Corporation, College Station, TX, USA).

## Results

### Literature search

**Figure 1:**
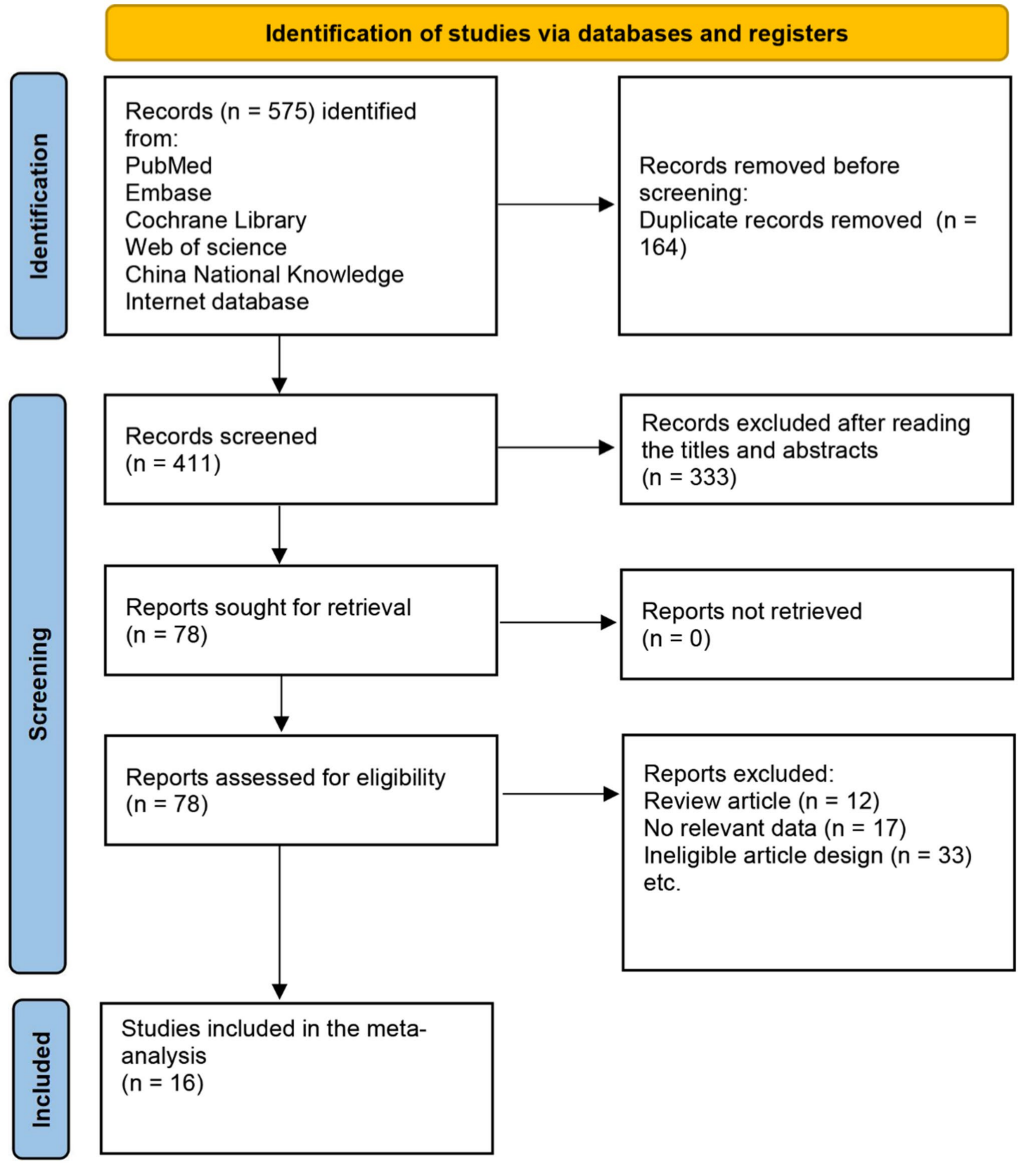
Flow diagram of the literature search and trial selection process. A total of 16 studies were incorporated from the 575 retrieved records.

The results of the study-selection process were shown in Figure 1. The primary search produced 575 records. After duplicates were removed, 411 studies were used to identify potentially relevant trials. After scanning titles and abstracts, we excluded 333 irrelevant articles. The remaining 78 full-text articles were reviewed, and 16 RCTs were included in the final meta-analysis, involving a total of 15,027 patients with IM or dysplasia (Wong et al., 2012; Choi et al., 2014; Cho et al., 2013; Correa et al., 2000; Leung et al., 2004; You et al., 2006; Fukase et al., 2008; Wong et al., 2004; Uemura et al., 1997; Saito et al., 2000; Saito et al., 2005; Yan et al., 2022; Choi et al., 2020; Choi et al., 2018; Li et al., 2019). A manual search of the reference lists of these studies did not yield any new eligible studies. The general characteristics of the included studies are presented in Table 1.

**Table 1:** Baseline characteristics of included trials.

Study	Country	Sample size	Mean age (yr)	Precursor status	Percentage male (%)	HP diagnosis	Number of GC	Intervention	Follow-up duration (years)
Correa et al. (2000)	Colombia	852	51.1	IM	46.1	HIS	5	Amoxicillin, metronidazole, and bismuth subsalicylate	6
Leung et al. (2004)	China	435	NA	IM	NA	RUT and HIS	10	Omeprazole, amoxicillin, and clarithromycin	5
You et al. (2006)	China	2,258	47.1	IM	50.4	Ser	46	Amoxicillin and omeprazole	9
Fukase et al. (2008)	Japan	544	68.5	Dysplasia	76.4	RUT or HIS	33	Lansoprazole, amoxicillin, and clarithromycin	3
Wong et al. (2004)	China	1,630	42.2	IM	54	RUT and HIS	18	Omeprazole, amoxicillin, clavulanate potassium, and metronidazole	7.5
Wong et al. (2012)	China	513	52.9	IM	46.2	UBT	4	Omeprazole, amoxicillin and clarithromycin	2
Choi et al. (2014)	Korea	901	60.4	Dysplasia	67.7	RUT or HIS	27	Omeprazole, amoxicillin, and clarithromycin	3
Cho et al. (2013)	Korea	169	56	IM	69.2	RUT or HIS	4	Rabeprazole, clarithromycin and amoxicillin	3
Uemura et al. (1997)	Japan	132	69	IM	72	Ser	6	Omeprazole and antimicrobial	3
Saito et al. (2000)	Japan	64	79.2	Dysplasia	54.7	Ser	4	Omeprazole and antimicrobial	2
Saito et al. (2005)	Japan	692	NA	Dysplasia	NA	Ser	5	Omeprazole and antimicrobial	4
Yan et al. (2022)	China	1,630	42.2	IM	54	RUT and HIS	56	Omeprazole, amoxicillin, clavulanate potassium, and metronidazole	26.5
Choi et al. (2018)	Korea	877	60.6	Dysplasia	67.7	RUT or HIS	54	Omeprazole, amoxicillin, and clarithromycin	6
Choi et al. (2020)	Korea	1,676	48.8	Dysplasia	49.5	RUT or HIS	33	Lansoprazole, amoxicillin, and clarithromycin	9.2
Choi et al. (2018)	Korea	396	59.8	Dysplasia	75.3	RUT or HIS	41	Amoxicillin, clavulanate potassium, and rabeprazole	5.9

GC, gastric cancer; IM, intestinal metaplasia; HP, Helicobacter pylori; HIS, histology; RUT, rapid urease test; Ser, serologic test; UBT, urea breath test.

### Study characteristics

Of the 16 included studies, six were conducted in China (Wong et al., 2012; Leung et al., 2004; You et al., 2006; Wong et al., 2004; Yan et al., 2022; Li et al., 2019), 4 in Japan (Fukase et al., 2008; Uemura et al., 1997; Saito et al., 2000; Saito et al., 2005), 5 in Korea (Choi et al., 2014; Cho et al., 2013; Choi et al., 2018; Choi et al., 2020), and the remaining 1 in Colombia (Correa et al., 2000). The follow-up duration for each trial ranged from 2.0 to 26.5 years, and 64–2,258 patients with IM or dysplasia were included in each trial. The mean age of the patients ranged from 42.2 to 79.2 years, and the percentage male ranged from 46.1 to 76.4%. Eight trials investigated the effect of *H. pylori* eradication in IM patients (Wong et al., 2012; Cho et al., 2013; Correa et al., 2000; Leung et al., 2004; You et al., 2006; Wong et al., 2004; Uemura et al., 1997; Yan et al., 2022), while the remaining 8 trials investigated patients with dysplasia (Choi et al., 2014; Fukase et al., 2008; Saito et al., 2000; Saito et al., 2005; Choi et al., 2018; Choi et al., 2020; Li et al., 2019). Study quality was evaluated using the Jadad scale (Table 2), and eight studies received a score of 5 (Wong et al., 2012; Cho et al., 2013; Correa et al., 2000; Fukase et al., 2008; Wong et al., 2004; Yan et al., 2022; Choi et al., 2020; Choi et al., 2018), 5 studies had a score of 4 (Choi et al., 2014; Leung et al., 2004; You et al., 2006; Li et al., 2019), 1 study had a score of 3 (Saito et al., 2005), and the remaining 2 studies had a score of 2 (Uemura et al., 1997; Saito et al., 2000).

**Table 2:** Quality scores of randomized controlled trials using Jadad scale.

Study	Randomization	Concealment of the treatment allocation	Blinding	Completeness of follow-up	Intention-to-treat analysis	Overall
Correa et al. (2000)	1	1	1	1	1	5
Leung et al. (2004)	1	0	1	1	1	4
You et al. (2006)	1	0	1	1	1	4
Fukase et al. (2008)	1	1	1	1	1	5
Wong et al. (2004)	1	1	1	1	1	5
Wong et al. (2012)	1	1	1	1	1	5
Choi et al. (2014)	1	0	1	1	1	4
Cho et al. (2013)	1	1	1	1	1	5
Uemura et al. (1997)	0	0	0	1	1	2
Saito et al. (2000)	0	0	0	1	1	2
Saito et al. (2005)	1	0	0	1	1	3
Yan et al. (2022)	1	1	1	1	1	5
Choi et al. (2018)	1	0	1	1	1	4
Choi et al. (2020)	1	1	1	1	1	5
Choi et al. (2018)	1	1	1	1	1	5
Li et al. (2019)	1	1	0	1	1	4

### *H. pylori* eradication on the risk of gastric cancer

**Figure 2:**
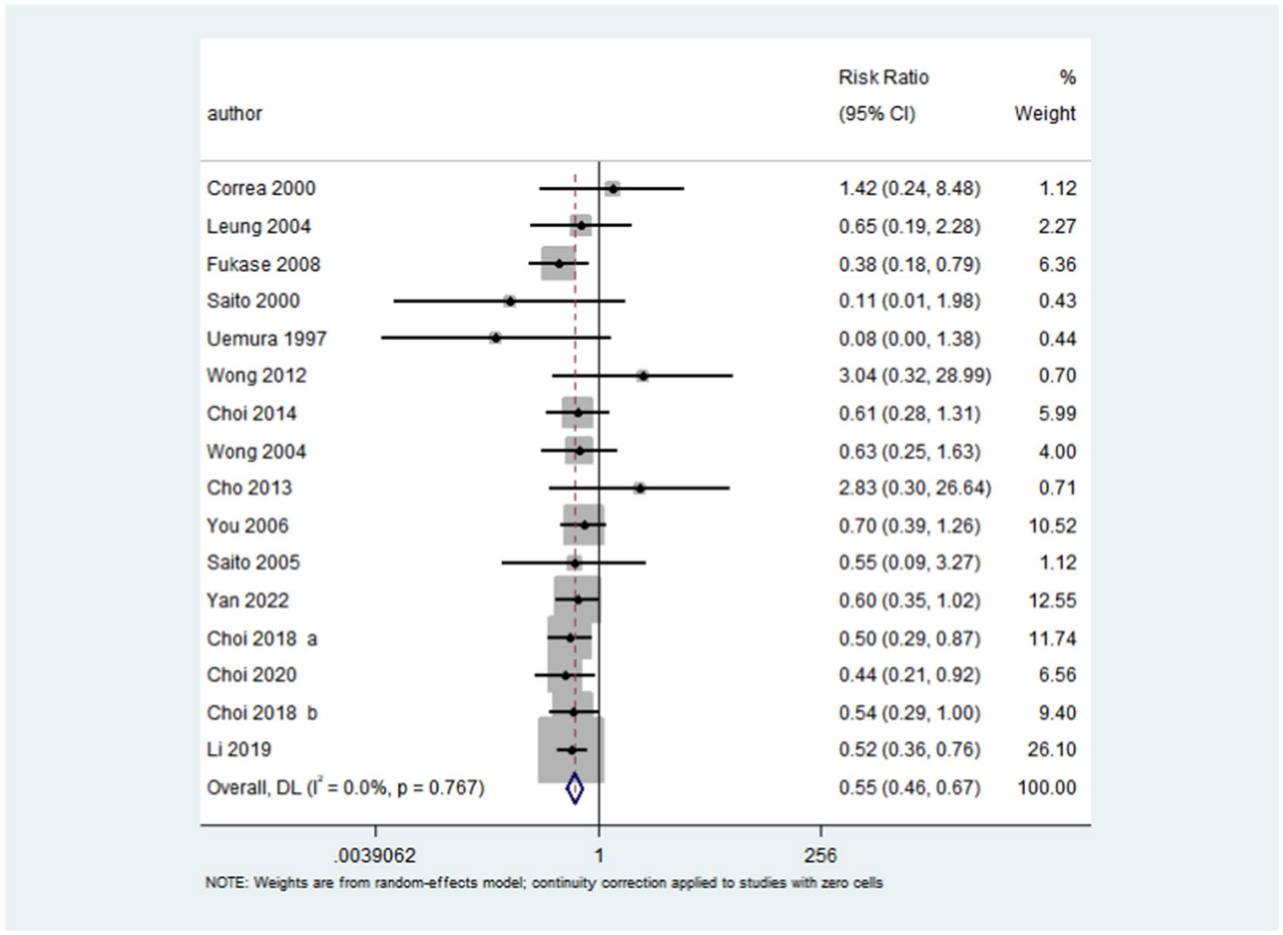
Effect of *H. pylori* eradication on the risk of gastric cancer in patients with IM or dysplasia. After pooling all the included trials, the summary RR was employed to assess the impact of *H. pylori* eradication on the risk of gastric cancer in patients with IM or dysplasia (RR: 0.55; 95% CI: 0.46–0.67; *p* < 0.001; I^2^ = 0.0%, *p*-value for heterogeneity: 0.767).

**Figure 3:**
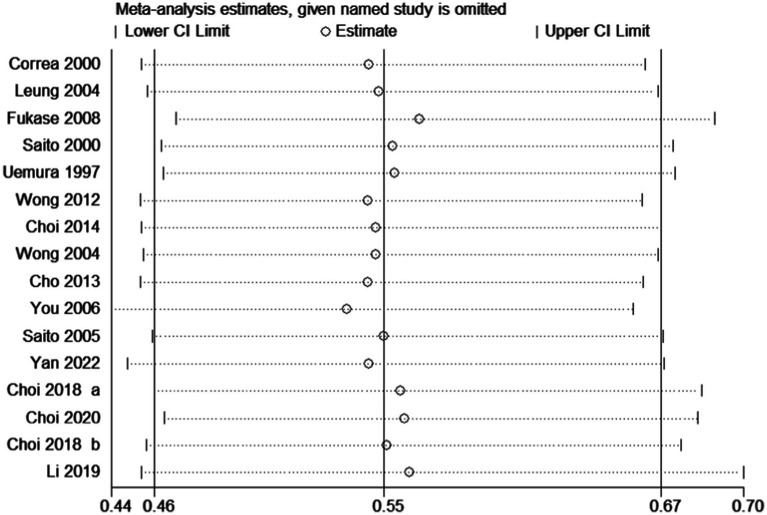
Sensitivity for the effect of *H. pylori* eradication on gastric cancer risk. Examine the impact on the overall conclusion following the exclusion of signle studies.

After pooling all included trials, the summary RR indicated that *H. pylori* eradication significantly reduced the risk of gastric cancer in patients with IM or dysplasia (RR: 0.55; 95% CI: 0.46–0.67; *p* < 0.001; I^2^ = 0.0%, *p*-value for heterogeneity: 0.767; Figure 2). Although no evidence of heterogeneity was observed, a sensitivity analysis was conducted to evaluate the influence of each single trial on the overall analysis. We noted that the conclusion was not affected following sequential exclusion of any study from the pooled analysis (Figure 3).

### Meta-regression and subgroup analysis

The meta-regression analysis was conducted according to publication year, sample size, mean age, percentage male, and follow-up duration. We noted that publication year (*p* = 0.424), sample size (*p* = 0.764), mean age (*p* = 0.375), percentage male (*p* = 0.936), and follow-up duration (*p* = 0.823) were not significant factors influencing the effect of *H. pylori* eradication on the risk of gastric cancer in patients with IM or dysplasia (Supplementary material). Subgroup analyses were conducted to evaluate the effect of *H. pylori* eradication on gastric cancer incidence in specific subgroup (Table 3). Overall, we noted no significant differences in gastric cancer risk between *H. pylori* eradication and no therapy if the study not conducted in other country, the sample size <500, percentage male <50.0%, follow-up duration <5.0 years, or if the study was of lower quality. *H. pylori* eradication was found to be associated with a reduced risk of gastric cancer in all other subsets.

**Table 3:** Subgroup analysis for gastric cancer risk.

Variable	Group	Number of trials	RR and 95% CI	*P-*value	*P*-value for heterogeneity
Country	Asia	15	0.55 (0.45–0.66)	<0.001	0.783
	Other	1	1.42 (0.24–8.48)	0.698	–
Publication year	After 2010	8	0.55 (0.44–0.68)	<0.001	0.890
	Before 2010	8	0.58 (0.37–0.90)	0.017	0.352
Sample size	≥500	11	0.56 (0.45–0.68)	<0.001	0.838
	<500	5	0.54 (0.26–1.11)	0.095	0.280
Mean age	≥60.0	5	0.60 (0.42–0.85)	0.004	0.430
	<60.0	9	0.53 (0.42–0.67)	<0.001	0.583
Percentage male (%)	≥50.0	11	0.55 (0.45–0.67)	<0.001	0.717
	< 50.0	3	0.86 (0.27–2.70)	0.797	0.169
Precursor status	IM	8	0.61 (0.40–0.93)	0.022	0.316
	Dysplasia	8	0.54 (0.43–0.67)	<0.001	0.934
HP diagnosis	HIS, RUT, or UBT	11	0.56 (0.43–0.72)	<0.001	0.726
	Ser	5	0.55 (0.42–0.73)	<0.001	0.434
Follow-up duration (years)	≥5.0	9	0.52 (0.42–0.64)	<0.001	0.836
	<5.0	7	0.69 (0.47–1.02)	0.063	0.553
Study quality	High	13	0.53 (0.43–0.65)	<0.001	0.788
	Low	3	0.74 (0.44–1.27)	0.280	0.470

RR, risk ratio; IM, intestinal metaplasia; HP, Helicobacter pylori; HIS, histology; RUT, rapid urease test; Ser, serologic test; UBT, urea breath test.

### Publication bias

**Figure 4:**
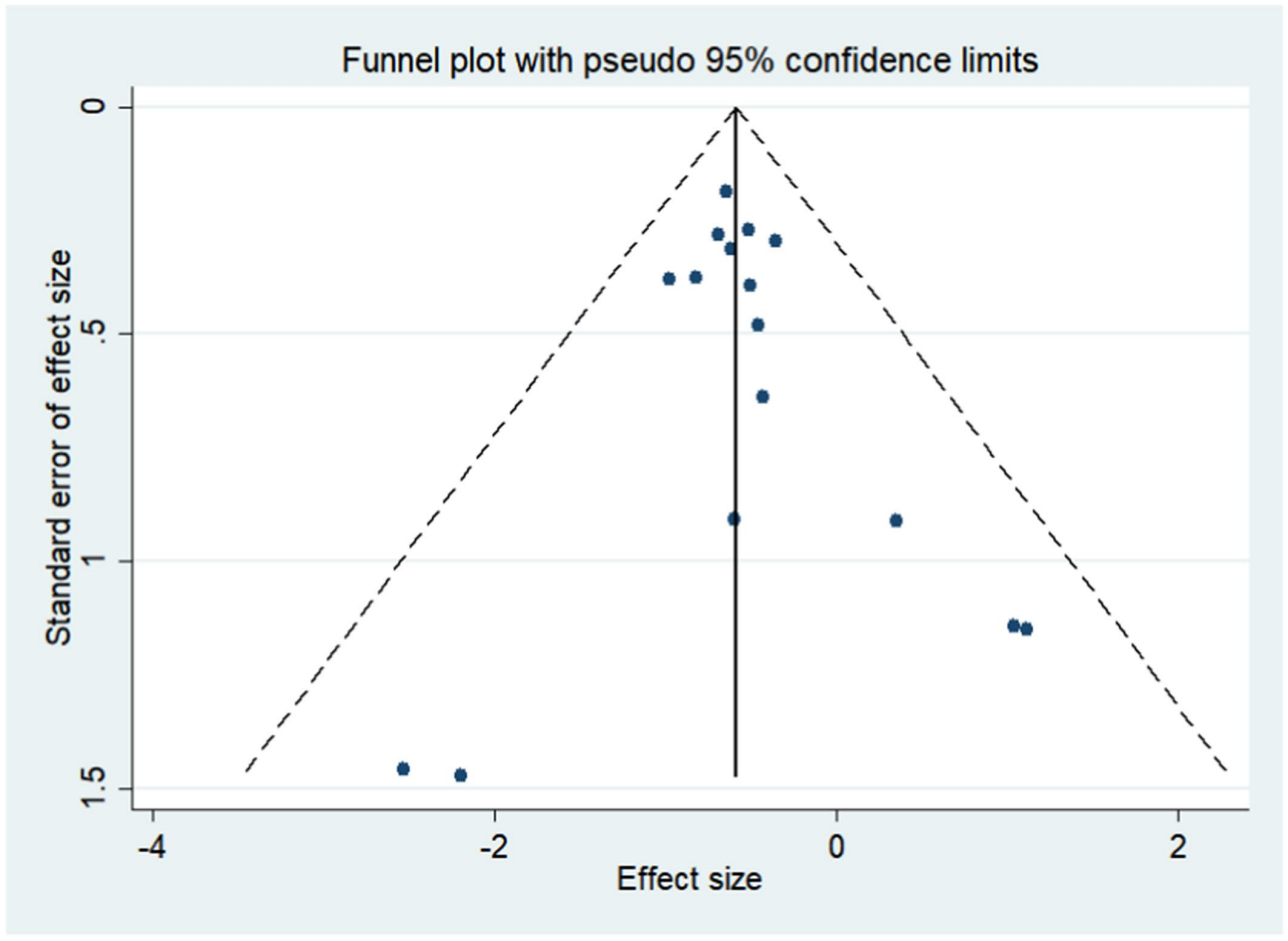
Funnel plot for the risk of gastric cancer. Publication bias for the outcome was evaluated using a funnel plot constructed from 16 studies.

The funnel plot distributed symmetrically around the pooled effect size, indicating that there was no significant publication bias in the included trials (Figure 4). The Egger’s and Begg’s test results showed no evidence of publication bias for gastric cancer (*p*-value for Egger: 0.573; *p*-value for Begg: 0.653).

## Discussion

Although there are numerous literature reports linking *H. pylori* with cancer, it is still important to integrate and analyze them to clarify definitive evidence of carcinogenesis.

The objective of the present meta-analysis was to determine the effect of *H. pylori* eradication on the risk of gastric cancer in patients with IM or dysplasia. Sixteen RCTs were identified involving 15,027 patients with IM or dysplasia. The summary results showed that *H. pylori* eradication was associated with a reduced risk of gastric cancer in these patients. Further, this relationship is differed according to country, sample size, percentage male, follow-up duration, and study quality. This finding will help to better define the risk of developing gastric cancer after *H. pylori* eradication in patients with IM or dysplasia, and could also help physicians select appropriate patients for *H. pylori* eradication in high-risk patients or patients already suffering from other conditions.

Eradication has been validated for preventing gastric cancer in patients with *H. pylori* infection. Fuccio et al. (2009) summarized seven RCTs and found that 1.1% of patients who received *H. pylori* eradication developed gastric cancer after follow-up, while 1.7% of untreated patients were diagnosed with gastric cancer. They indicated that patients receiving *H. pylori* eradication had an RR for gastric cancer of 0.65 (95% CI: 0.43–0.98) as compared with the untreated group. Furthermore, Jung et al. (2015) demonstrated that *H. pylori* eradication was associated with a lower risk of metachronous lesions following endoscopic resection of gastric neoplasms (OR: 0.392; *p* < 0.001). This treatment effect persisted after adjusted *H. pylori* eradication. Tan et al. (2015) combined 16 cohort studies and suggested that *H. pylori* eradication reduced the incidence of gastroesophageal reflux disease by 13%, but this association was not statistically significant. They further indicated that *H. pylori* eradication should be pursued due to the association of *H. pylori* infection with higher risks of acute and chronic gastritis and peptic ulcer diseases. Chen et al. (2016) conducted a meta-analysis based on 10 RCTs and found *H. pylori* treatment has no significant effect on the risk of gastric cancer in patients with intestinal metaplasia or dysplasia. Further, they point out the summary risk for gastric cancer main focused in patients with non-atrophic gastritis, atrophic gastritis. However, several studies focused on patients with other characteristics. Further, whether *H. pylori* eradication is protective against gastric cancer in patients already diagnosed with IM or dysplasia according to different characteristics remained controversial. As a result, we conducted this updated meta-analysis of RCTs to further clarify the effect that *H. pylori* eradication has on preventing gastric cancer in patients with IM or dysplasia.

In recent years, several RCTs have evaluated the effect of *H. pylori* eradication on the risk of gastric cancer in patients with IM or dysplasia. Zhu et al. (2023) included 15 studies and found that compared with the control group, *H. pylori* eradication could significantly prevent the progression of precancerous lesions of gastric cancer and reverse them. However, there are notable differences between the findings of Zhu et al. (2023) and our study. Zhu et al. (2023) reported that *H. pylori* eradication significantly inhibited the progression of intestinal metaplasia (RR = 0.80, 95% CI: 0.69–0.94, *p* < 0.01), which is consistent with our results. However, according to Zhu et al. (2023), *H. pylori* eradication did not demonstrate a significant advantage in preventing atypical hyperplasia (RR = 0.86, 95% CI: 0.37–2.00) or improving its progression (RR = 0.89, 95% CI: 0.47–1.70). These discrepancies may be attributed to the differing inclusion criteria used in the two studies. Wong et al. (2012) included 1,024 patients with IM and found that celecoxib or *H. pylori* eradication both had beneficial effects on the regression of advanced gastric lesions, yet had no significant effect on the risk of gastric cancer. Choi et al. (2014) evaluated the effects of *H. pylori* eradication on the incidence of metachronous carcinoma after endoscopic resection of gastric tumors. They did not find a significant difference between the groups in metachronous gastric carcinoma. Cho et al. (2013) included 190 IM patients and demonstrated that *H. pylori* eradication could benefit gastric cancer patients due to its association with lower atrophy and IM at 36 months following subtotal gastrectomy, yet, again, the decreased incidence of gastric cancer was not statistically significant. This could be due to the lower-than-expected incidence of gastric cancer, resulting in broad 95% CIs leading to a statistically insignificant difference. Furthermore, the sample size was smaller than expected, and the trials were designed with atrophy and IM lesions as primary end points instead of gastric cancer, which might also account for the lack of statistical difference. Finally, different baseline patient characteristics or differences in treatment strategies might introduce uncontrolled biases and influence the effects of *H. pylori* eradication on the risk of gastric cancer.

The subgroup analysis indicated that *H. pylori* eradication significantly reduced the risk of gastric cancer in multiple subsets, yet some subsets saw no effect. First, we noted that *H. pylori* eradication had no significant effect on gastric cancer if the study was not conducted in Asia (Colombia). This could be a result of the majority of the included trials being conducted in Asia, which has a higher incidence of gastric cancer than Western countries (Torre et al., 2015), possibly resulting from differences in dietary patterns (Bertuccio et al., 2013). This lack of significant difference could also simply result from there being only a single trial included in this subset (Correa et al., 2000). Second, there was no significant difference for the effect of *H. pylori* eradication on gastric cancer if the sample size was <500. A possible reason for this could be that the incidence of gastric cancer was lower in general for studies with lower sample sizes. Third, the treatment effect of *H. pylori* eradication is main focused in male patients. The possible reason for this could be male with higher incidence of gastric cancer. Fourth, in the studies where the follow-up duration was <5 years, there was no significant difference in the risk of gastric cancer between patients with *H. pylori* eradication and those without treatment. This might be attributed to the insufficiently long follow-up period, during which the lesions had not yet advanced to gastric cancer. Finally, when combining lower quality trials, no significant difference was detected for gastric cancer risk between *H. pylori*-eradicated patients and untreated patients. Although most trials in this lower quality subset reported satisfactory follow-up completeness and use of intention-to-treat analysis, uncontrolled biases may have been introduced in their randomization, concealment of the treatment allocation, and blinding. Therefore, any results based on these lower quality trials should be evaluated critically before making recommendations for patients with IM or dysplasia.

A few advantages of this study should be highlighted: (1) Only RCTs were included for evaluation, which could eliminate potential overestimations of the treatment effect size seen in observational studies; (2) The treatment effect of *H. pylori* eradication in patients with IM or dysplasia for preventing gastric cancer was quantitatively analyzed based on a large sample size, and thus our findings are potentially more robust than are those of any individual study; and (3) The treatment effect of *H. pylori* eradication was evaluated according to different characteristics and compared with corresponding subsets, providing relative guides for future studies.

As with many meta-analyses, several limitations should be mentioned. First, a language bias may exist in selecting RCTs published only in English. Secondly, all the included studies were conducted in Asia except for one. If the study was not carried out in Asia (Colombia), the eradication of *H. pylori* had no significant effect on gastric cancer. Therefore, the results of this study are mainly applicable to research in Asia. Third, different study qualities and sample sizes between the included trials could influence the data and consequently introduce uncontrollable biases. Fourth, the analysis used pooled data, and individual data were not available, restricting us from performing a more detailed relevant analysis and obtaining more comprehensive results. Finally, due to the varying lengths of follow-up periods across different studies, this heterogeneity can significantly influence the reported outcomes. Moreover, differences in follow-up durations are a critical factor contributing to between-study heterogeneity. Therefore, when conducting a meta-analysis, it is essential to consider the disparities in average follow-up times among studies. However, in our study, the included research spans a wide range, with follow-up periods ranging from 2 to 26.5 years. Incorporating the average follow-up time into the analysis would introduce considerable bias due to this variability. Consequently, we did not include the average follow-up time in our calculations. Compared with the study by Hahn et al. (2024), this represents a significant limitation of our study. In future research, we will incorporate the average follow-up time as an inclusion criterion to standardize patient follow-up periods.

In this updated meta-analysis, we note that *H. pylori* eradication shows a beneficial effect against the progression of gastric cancer when compared with untreated patients. As the goal of gastric cancer prevention is to minimize gastric cancer incidence and enhance quality of life, *H. pylori* eradication should be recommended for high-risk patients. Additional large-scale trials investigating the primary prevention of gastric cancer in asymptomatic *H. pylori* infection patients must be further explored.

## Data Availability

The original contributions presented in the study are included in the article/Supplementary material, further inquiries can be directed to the corresponding authors.

## References

[ref1] Ades A. E. Lu G. Higgins J. P. (2005). The interpretation of random-effects meta analysis in decision models. Med. Decis. Mak. 25, 646–654. doi: 10.1177/0272989X05282643, PMID: 16282215

[ref2] Asaka M. Kato M. Takahashi S. Fukuda Y. Sugiyama T. Ota H. . (2010). Guidelines for the management of *Helicobacter pylori* infection in Japan. Helicobacter 15, 1–20. doi: 10.1111/j.1523-5378.2009.00738.x, PMID: 20302585

[ref3] Begg C. B. Mazumdar M. (1994). Operating characteristics of a rank correlation test for publication bias. Biometrics 50, 1088–1101. doi: 10.2307/2533446, PMID: 7786990

[ref4] Bertuccio P. Rosato V. Andreano A. Ferraroni M. Decarli A. Edefonti V. . (2013). Dietary patterns and gastric cancer risk: a systematic review and meta-analysis. Ann. Oncol. 24, 1450–1458. doi: 10.1093/annonc/mdt108, PMID: 23524862

[ref5] Chen H. N. Wang Z. Li X. Zhou Z. G. (2016). *Helicobacter pylori* eradication cannot reduce the risk of gastric cancer in patients with intestinal metaplasia and dysplasia: evidence from a meta-analysis. Gastric Cancer 19, 166–175. doi: 10.1007/s10120-015-0462-7, PMID: 25609452

[ref6] Cho S. J. Choi I. J. Kook M. C. Yoon H. Park S. Kim C. G. . (2013). Randomised clinical trial: the effects of *Helicobacter pylori* eradication on glandular atrophy and intestinal metaplasia after subtotal gastrectomy for gastric cancer. Aliment. Pharmacol. Ther. 38, 477–489. doi: 10.1111/apt.12402, PMID: 23822578

[ref7] Choi I. J. Chan G. K. Lee J. Y. Kim Y-I. Kook M-C. Park B. . (2020). Family history of gastric Cancer and *Helicobacter pylori* treatment. N. Engl. J. Med. 382, 427–436. doi: 10.1056/NEJMoa1909666, PMID: 31995688

[ref8] Choi J. M. Kim S. G. Choi J. Park J. Y. Oh S. Yang H. J. . (2018). Effects of *Helicobacter pylori* eradication for metachronous gastric cancer prevention: a randomized controlled trial. Gastrointest. Endosc. 88, 475–485. doi: 10.1016/j.gie.2018.05.009, PMID: 29800546

[ref9] Choi I. J. Kook M. C. Kim Y. I. Cho S. J. Lee J. Y. Kim C. G. . (2018). *Helicobacter pylori* therapy for the prevention of Metachronous gastric Cancer. N. Engl. J. Med. 378, 1085–1095. doi: 10.1056/NEJMoa1708423, PMID: 29562147

[ref10] Choi J. Sang G. K. Yoon H. Im J. P. Kim J. S. Kim W. H. . (2014). Eradication of *Helicobacter pylori* after endoscopic resection of gastric tumors does not reduce incidence of Metachronous gastric carcinoma. Clin. Gastroenterol. Hepatol. 12, 793–800.e1. doi: 10.1016/j.cgh.2013.09.057 24100112

[ref11] Correa P. (1984). Chronic gastritis as a cancer precursor. Scand. J. Gastroenterol. Suppl. 104, 131–136, PMID: 6597545

[ref12] Correa P. (1988). A human model of gastric carcinogenesis. Cancer Res. 48, 3554–3560, PMID: 3288329

[ref13] Correa P. Fontham E. T. Bravo J. C. Bravo L. E. Ruiz B. Zarama G. . (2000). Chemoprevention of gastric dysplasia: randomized trial of antioxidant supplements and anti-*Helicobacter pylori* therapy. J. Natl. Cancer Inst. 92, 1881–1888. doi: 10.1093/jnci/92.23.1881, PMID: 11106679

[ref14] Correa P. Fox J. Fontham E. Ruiz B. Lin Y. Zavala D. . (1990). Helicobacter pyloriand gastric carcinoma. Serum antibody prevalence in populations with contrasting cancer risks. Cancer 66, 2569–2574. doi: 10.1002/1097-0142(19901215)66:12<2569::AID-CNCR2820661220>3.0.CO;2-I, PMID: 2249197

[ref15] Deeks J. J. Altman D. G. Bradburn M. J. (2001). “Statistical methods for examining heterogeneity and combining results from several studies in meta-analysis” in Systematic reviews in health care: Metaanalysis in context. eds. Egger M. Davey Smith G. Altman D. G.. 2nd ed (London: BMJ Books), 285–312.

[ref16] Deeks J. J. Higgins J. P. Altman D. G. (2008). “Analyzing data and undertaking meta-analyses” in Cochrane handbook for systematic reviews of interventions 5.0.1. eds. Higgins J. Green S. (Oxford: The Cochrane Collaboration).

[ref17] DerSimonian R. Laird N. (1986). Meta-analysis in clinical trials. Control. Clin. Trials 7, 177–188. doi: 10.1016/0197-2456(86)90046-2 3802833

[ref18] Egger M. Davey Smith G. Schneider M. Minder C. (1997). Bias in meta-analysis detected by a simple, graphical test. BMJ 315, 629–634. doi: 10.1136/bmj.315.7109.629, PMID: 9310563 PMC2127453

[ref19] Ferlay J. Shin H. R. Bray F. Forman D. Mathers C. Parkin D. M. (2010). Estimates of worldwide burden of cancer in 2008: GLOBOCAN 2008. Int. J. Cancer 127, 2893–2917. doi: 10.1002/ijc.25516, PMID: 21351269

[ref20] Ferlay J. Soerjomataram I. Ervik M. Dikshit R. Eser S. Mathers C. . (2013). GLOBOCAN 2012 v1.0, Cancer incidence and mortality worldwide: IARC CancerBase no. 11 [internet]. Lyon: International Agency for Research on Cancer.

[ref21] Fuccio L. Zagari R. M. Eusebi L. H. Laterza L. Cennamo V. Ceroni L. . (2009). Meta-analysis: can *Helicobacter pylori* eradication treatment reduce the risk for gastric cancer? Ann. Intern. Med. 151, 121–128. doi: 10.7326/0003-4819-151-2-200907210-00009, PMID: 19620164

[ref22] Fukase K. Kato M. Kikuchi S. Inoue K. Uemura N. Okamoto S. . (2008). Effect of eradication of *Helicobacter pylori* on incidence of metachronous gastric carcinoma after endoscopic resection of early gastric cancer: an open-label, randomized controlled trial. Lancet 372, 392–397. doi: 10.1016/S0140-6736(08)61159-9, PMID: 18675689

[ref23] Hahn A. I. Mülder D. T. Huang R. J. Zhou M. J. Blake B. Omofuma O. . (2024). Global progression rates of precursor lesions for gastric Cancer: a systematic review and Meta-analysis. Clin. Gastroenterol. Hepatol. 22, S1542–S3565. doi: 10.1016/j.cgh.2024.09.003, PMID: 39362617 PMC11958785

[ref24] Higgins J. P. Thompson S. G. Deeks J. J. Altman D. G. (2003). Measuring inconsistency in meta-analyses. BMJ 327, 557–560. doi: 10.1136/bmj.327.7414.557, PMID: 12958120 PMC192859

[ref25] Huang J. Q. Zheng G. F. Sumanac K. Irvine E. J. Hunt R. H. (2003). Meta-analysis of the relationship between CagA seropositivity and gastric cancer. Gastroenterology 125, 1636–1644. doi: 10.1053/j.gastro.2003.08.033, PMID: 14724815

[ref26] International Agency for Research on Cancer (1994). IARC monographs on the evaluation of carcinogenic risks TO humans. Schistosomes, liver flukes and *Helicobacter pylori* volume 61IARC. Lyon: International Agency for Research on Cancer, 177–240. PMC7681621 7715068

[ref27] Jadad A. R. Moore R. A. Carroll D. Jenkinson C. Reynolds D. J. M. Gavaghan D. J. . (1996). Assessing the quality of reports of randomized clinical trials: is blinding necessary? Control. Clin. Trials 17, 1–12. doi: 10.1016/0197-2456(95)00134-4, PMID: 8721797

[ref28] Jung D. H. Kim J. H. Chung H. S. Park J. C. Shin S. K. Lee S. K. . (2015). *Helicobacter pylori* eradication on the prevention of metachronous lesions after endoscopic resection of gastric neoplasm: a meta-analysis. PLoS One 10:e0124725. doi: 10.1371/journal.pone.0124725, PMID: 25915048 PMC4411104

[ref29] Lee Y. C. Chiang T. H. Chou C. K. Tu Y. K. Liao W. C. Wu M. S. . (2016). Association between *Helicobacter pylori* eradication and gastric Cancer incidence: a systematic review and Meta-analysis. Gastroenterology 150, 1113–1124. doi: 10.1053/j.gastro.2016.01.028, PMID: 26836587

[ref30] Leung W. K. Lin S. R. Ching J. Y. To K. F. Ng E. K. Chan F. K. . (2004). Factors predicting progression of gastric intestinal metaplasia: results of a randomised trial on *Helicobacter pylori* eradication. Gut 53, 1244–1249. doi: 10.1136/gut.2003.034629, PMID: 15306578 PMC1774213

[ref31] Li W. Zhang J. Ma J. Li Z-X. Zhang L. Zhang Y. . (2019). Effects of *Helicobacter pylori* treatment and vitamin and garlic supplementation on gastric cancer incidence and mortality: follow-up of a randomized intervention trial. BMJ 366:l5016. doi: 10.1136/bmj.l5016, PMID: 31511230 PMC6737461

[ref32] Maeda S. Yoshida H. Ogura K. Yamaji Y. Ikenoue T. Mitsushima T. . (2000). Assessment of gastric carcinoma risk associated with *Helicobacter pylori*may vary depending on the antigen used: CagA specific enzyme-linked immunoadsorbent assay (ELISA) versus commercially available *H. pylori*elisas. Cancer 88, 1530–1535. doi: 10.1002/(SICI)1097-0142(20000401)88:7<1530::AID-CNCR5>3.0.CO;2-4, PMID: 10738209

[ref33] Malfertheiner P. Megraud F. O’Morain C. A. Atherton J. Axon A. T. R. Bazzoli F. . (2012). Management of *Helicobacter pylori* infection-the Maastricht IV/Florence consensus report. Gut 61, 646–664. doi: 10.1136/gutjnl-2012-302084 22491499

[ref34] Moher D. Liberati A. Tetzlaff J. Altman D. G. (2010). Preferred reporting items for systematic reviews and meta-analyses: the PRISMA statement. Int. J. Surg. 8, 336–341. doi: 10.1016/j.ijsu.2010.02.007 20171303

[ref35] Parsonnet J. Friedman G. D. Vandersteen D. P. Chang Y. Vogelman J. H. Orentreich N. . (1991). *Helicobacter pylori* infection and the risk of gastric carcinoma. N. Engl. J. Med. 325, 1127–1131. doi: 10.1056/NEJM199110173251603 1891020

[ref36] Saito K. Arai K. Mori M. Kobayashi R. Ohki I. (2000). Effect of *Helicobacter pylori* eradication on malignant transformation of gastric adenoma. Gastrointest. Endosc. 52, 27–32. doi: 10.1067/mge.2000.106112, PMID: 10882958

[ref37] Saito D. Boku N. Fujioka T. Fukuda Y. (2005). Impact of *H. pylori* eradication on gastric cancer prevention: endoscopic results of the Japanese intervention trial. Gastroenterology 128:A4.

[ref38] Tan J. Wang Y. Sun X. Cui W. Ge J. Lin L. (2015). The effect of *Helicobacter pylori* eradication therapy on the development of gastroesophageal reflux disease. Am J Med Sci 349, 364–371. doi: 10.1097/MAJ.0000000000000429, PMID: 25767896

[ref39] Thompson S. G. Higgins J. P. (2002). How should meta-regression analyses be undertaken and interpreted? Stat. Med. 21, 1559–1573. doi: 10.1002/sim.1187 12111920

[ref40] Tobias A. (1999). Assessing the influence of a single study in meta-analysis. Stata Tech. Bull. 47, 15–17.

[ref41] Torre L. A. Bray F. Siegel R. L. Ferlay J. Lortet-Tieulent J. Jemal A. (2015). Global cancer statistics, 2012. CA Cancer J. Clin. 65, 87–108. doi: 10.3322/caac.21262 25651787

[ref42] Uemura N. Mukai T. Okamoto S. Yamaguchi S. Mashiba H. Taniyama K. . (1997). Effect of *Helicobacter pylori* eradication on subsequent development of cancer after endoscopic resection of early gastric cancer. Cancer Epidemiol. Biomarkers Prev. 6, 639–642. 9264278

[ref43] Uemura N. Okamoto S. Yamamoto S. Matsumura N. Yamaguchi S. Yamakido M. . (2001). *Helicobacter pylori* infection and the development of gastric cancer. N. Engl. J. Med. 345, 784–789. doi: 10.1056/NEJMoa001999, PMID: 11556297

[ref44] Wong B. C. Lam S. K. Wong W. M. Chen J. S. Zheng T. T. Feng R. E. . (2004). *Helicobacter pylori* eradication to prevent gastric cancer in a high-risk region of China: a randomized controlled trial. JAMA 291, 187–194. doi: 10.1001/jama.291.2.187, PMID: 14722144

[ref45] Wong B. C. Zhang L. Ma J. Pan K-f. Li J-y. Shen L. . (2012). Effects of selective COX-2 inhibitor and *Helicobacter pylori* eradication on precancerous gastric lesions. Gut 61, 812–818. doi: 10.1136/gutjnl-2011-300154, PMID: 21917649

[ref46] Yan L. Chen Y. Chen F. Tao T. Hu Z. Wang J. . (2022). Effect of *Helicobacter pylori* eradication on gastric Cancer prevention: updated report from a randomized controlled trial with 26.5 years of follow-up. Gastroenterology 163, 154–162. doi: 10.1053/j.gastro.2022.03.039, PMID: 35364066

[ref47] You W. Brown L. M. Zhang L. Li J. Y. Jin M. L. Chang Y. S. . (2006). Randomized double-blind factorial trial of three treatments to reduce the prevalence of precancerous gastric lesions. J. Natl. Cancer Inst. 98, 974–983. doi: 10.1093/jnci/djj264, PMID: 16849680

[ref48] Zhu F. Zhang X. Li P. Zhu Y. (2023). Effect of *Helicobacter pylori* eradication on gastric precancerous lesions: a systematic review and meta-analysis. Helicobacter 28:e13013. doi: 10.1111/hel.13013, PMID: 37602719

