# Supplementary material for: Effect of Helicobacter pylori eradication on gastric cancer risk in patients with intestinal metaplasia or dysplasia: a meta-analysis of randomized controlled trials
Source: Front Microbiol. 2025 Mar 12;16:1530549. doi: 10.3389/fmicb.2025.1530549 (PMC11938427; doi:10.3389/fmicb.2025.1530549)
Supplement: Supplementary file 1 [file Data_Sheet_1.doc]

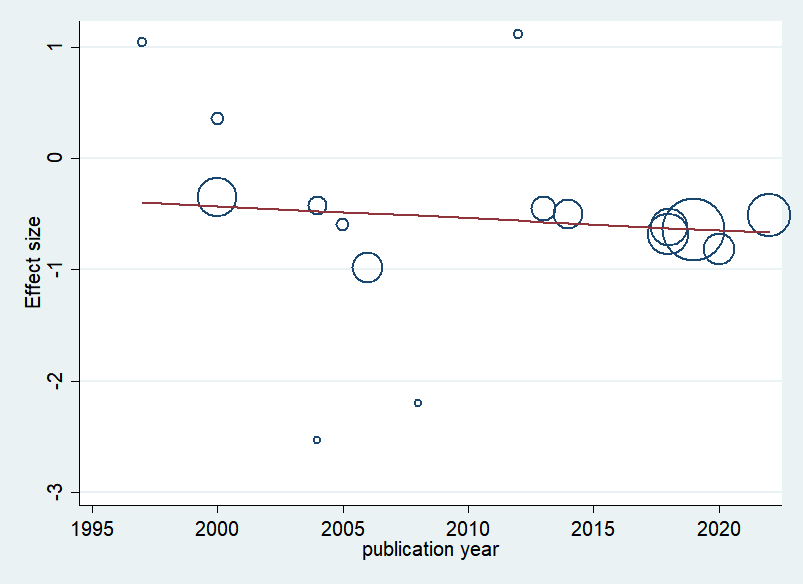


Figure S1. Meta-regression of gastric cancer based on publication year (P=0.424)


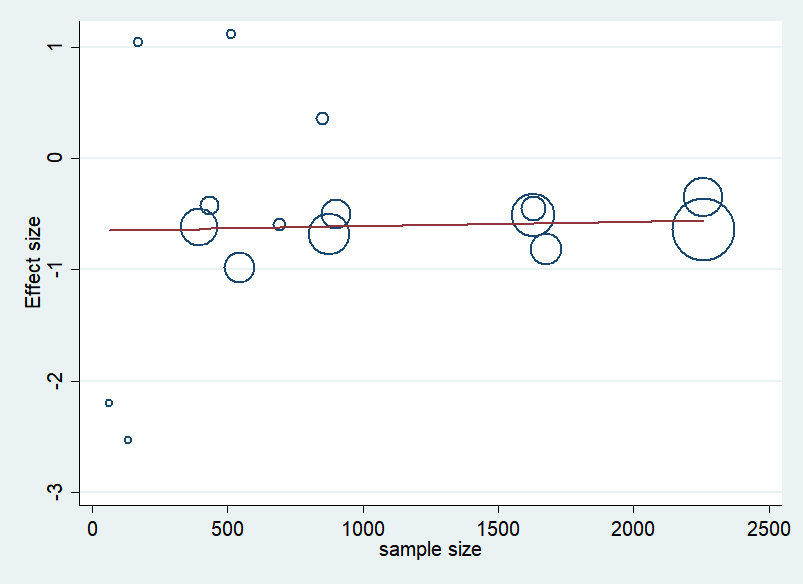


Figure S2. Meta-regression of gastric cancer based on sample size (P=0.764)


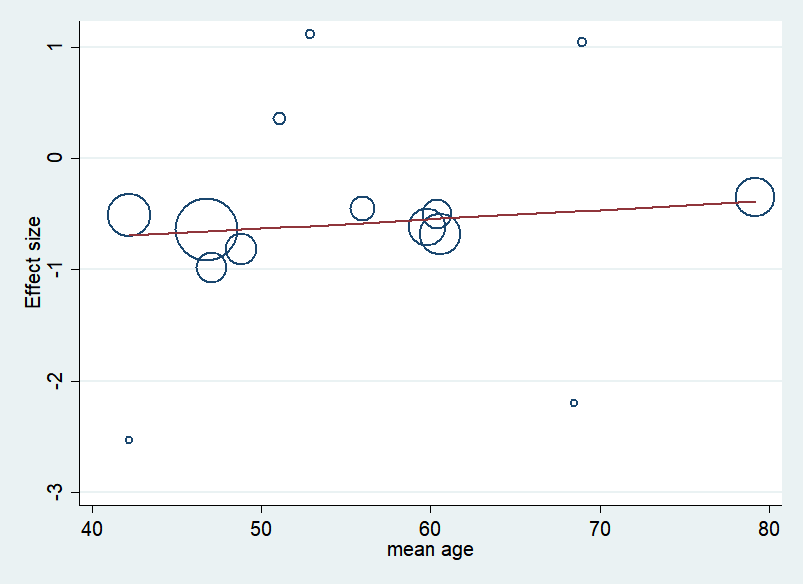


Figure S3. Meta-regression of gastric cancer based on mean age (P=0.375)


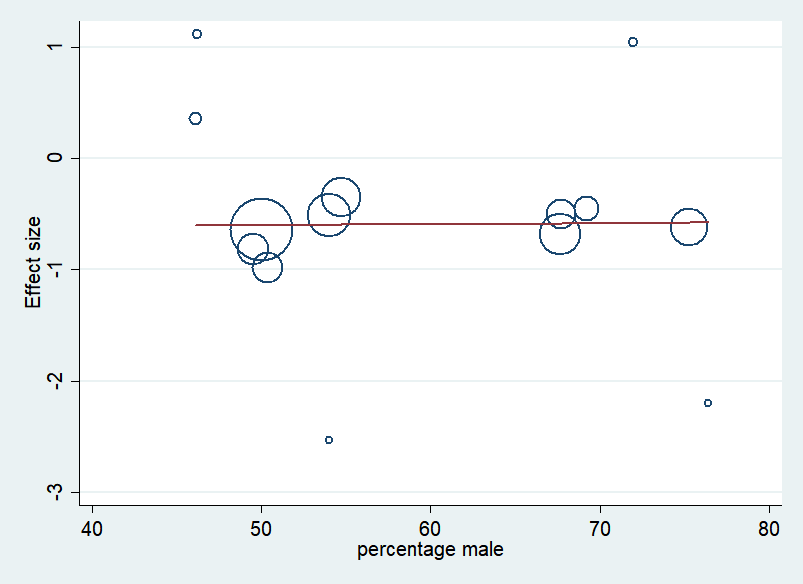


Figure S4. Meta-regression of gastric cancer based on percentage male (P=0.936)


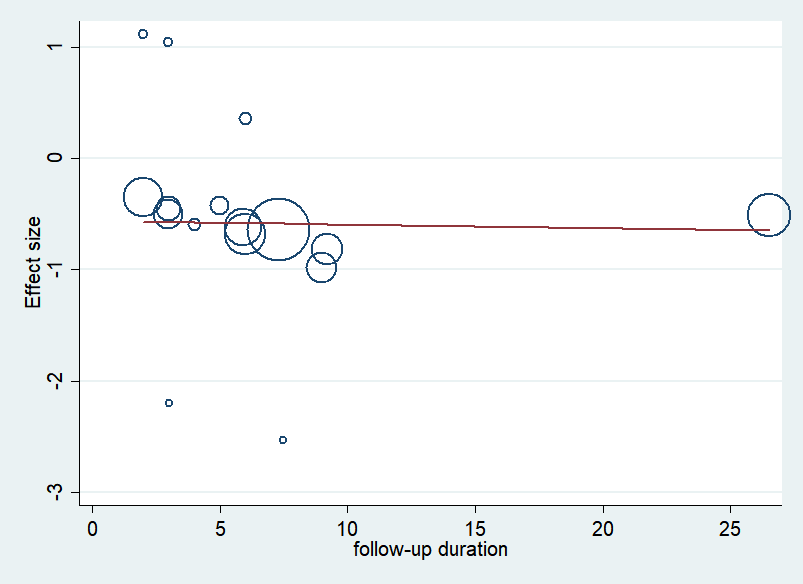


Figure S5. Meta-regression of gastric cancer based on follow-up duration (P=0.823)
